# Soft tissue uptake of 99mTc-MDP in a case of myxoid liposarcoma with breast metastasis

**DOI:** 10.11604/pamj.2016.25.184.9382

**Published:** 2016-11-22

**Authors:** Abdelhamid Biyi, Abderrahim Doudouh

**Affiliations:** 1 Department of Nuclear Medicine, Mohammed V Military Teaching Hospital, Mohammed V University of Rabat, Morocco

**Keywords:** Bone scintigraphy, soft tissue uptake, myxoid liposarcoma, breast metastasis

## Image in medicine

Breast metastasis arising from non mammary malignancy is very rare and occurs in malignant melanomas, sarcomas, lung cancers, ovarian tumors, renal and thyroid carcinomas. Few cases of breast metastasis from myxoid liposarcoma have been reported in the worldwide literature. We report herein a case of a 51-year-old woman with 2-year history of a slowly growing, painless mass of the right proximal thigh. Imaging investigations showed an intermuscular mass with low-attenuation areas on the CT scan (A) and high signal intensity on T2-weighted MR images (B). Material obtained by biopsy revealed a myxoid liposarcoma. A bone scan performed as part of the initial staging process showed multiple hot spots suggestive of skeletal metastasis in addition to increased soft tissue tracer uptake projected over the right proximal thigh and the left mammary gland corresponding respectively to the primitive tumour and a breast metastasis (C). Chest CT scan showed a left breast mass with similar attenuation feature to the primitive tumour (D). The patient was treated by six chemotherapy cycles (Doxorubicine, Ifosfamide) with good control of her disease on tree years follow up (E). Myxoid Liposarcoma is a rare soft-tissue tumour with a possible aggressive evolution leading to intensive chemotherapy protocols. Soft tissue uptake on bone scan is exceptional, but can occur in a large spectrum of disease. In this case, necrosis and high-water content of myxoid tissue, reflected as marked high signal intensity on T2-weighted MR images, may contribute to the soft tissue uptake mechanism of the bone seeking radiopharmaceutical.

**Figure 1:**
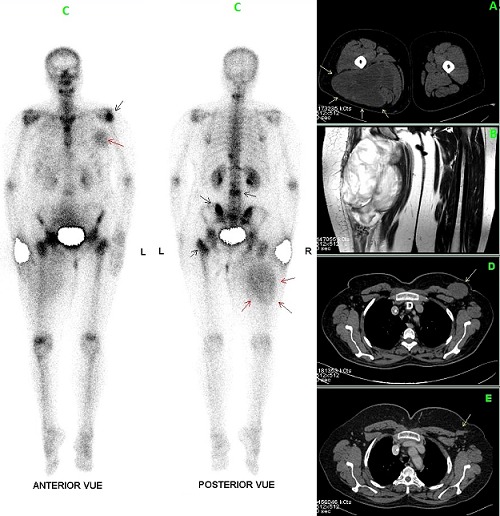
A) intermuscular mass with low-attenuation areas (white arrows) of the right proximal thigh on the CT scan; B) intermuscular mass with marked high signal intensity on T2-weighted MR images, and marked high signal intensity on T2-weighted MR images; C) bone scintigraphy showing multiple hot spots over the proximal epiphysis of the left humerus, sternum, T10-12 and L2-3 and S1 vertebrae, suggestive of skeletal metastases (black arrows) in addition to a soft tissue uptake projected over the right proximal thigh and the left mammary gland corresponding respectively to the primitive tumour (red arrows) and a breast metastasis (red arrow); D) left breast metastasis revealed as a low attenuation area (white arrow) on chest computerised tomography; E) regression of the breast metastasis (white arrow) on a follow-up CT tree years later

